# Impairment of neuronal mitochondrial function by l-DOPA in the absence of oxygen-dependent auto-oxidation and oxidative cell damage

**DOI:** 10.1038/s41420-021-00547-4

**Published:** 2021-06-28

**Authors:** Philipp Hörmann, Sylvie Delcambre, Jasmin Hanke, Robert Geffers, Marcel Leist, Karsten Hiller

**Affiliations:** 1 Department of Bioinformatics and Biochemistry, Technische Universität Braunschweig, Braunschweig, Germany; 2 Luxembourg Centre for Systems Biomedicine, University of Luxembourg, Esch-sur-Alzette, Luxembourg; 3 Genome Analytics, Helmholtz-Center for Infection Research, Braunschweig, Germany; 4 In Vitro Toxicology and Biomedicine, University of Konstanz, Konstanz, Germany

**Keywords:** Medical research, Parkinson's disease

## Abstract

L-3,4-Dihydroxyphenylalanin (l-DOPA or levodopa) is currently the most used drug to treat symptoms of Parkinson’s disease (PD). After crossing the blood–brain barrier, it is enzymatically converted to dopamine by neuronal cells and restores depleted endogenous neurotransmitter levels. l-DOPA is prone to auto-oxidation and reactive intermediates of its degradation including reactive oxygen species (ROS) have been implicated in cellular damage. In this study, we investigated how oxygen tension effects l-DOPA stability. We applied oxygen tensions comparable to those in the mammalian brain and demonstrated that 2% oxygen almost completely stopped its auto-oxidation. l-DOPA even exerted a ROS scavenging function. Further mechanistic analysis indicated that l-DOPA reprogrammed mitochondrial metabolism and reduced oxidative phosphorylation, depolarized the mitochondrial membrane, induced reductive glutamine metabolism, and depleted the NADH pool. These results shed new light on the cellular effects of l-DOPA and its neuro-toxicity under physiological oxygen levels that are very distinct to normoxic in vitro conditions.

## Introduction

Parkinson’s disease (PD) is the second most common neurodegenerative disease after Alzheimer’s disease (AD) and affects ~1% of the worldwide population with an age over 65 with raising trend [1, 2]. It is characterized by a substantial loss of dopaminergic neurons within the *substantia nigra* in the mid brain and results in lower levels of the neurotransmitter dopamine (DA) which is responsible for the classic symptoms like tremor, bradykinesia, rigidity and postural instability [3]. PD patients were firstly treated with levodopa (l-DOPA) in 1961 [4]. In contrast to DA it is able to cross the blood–brain barrier (BBB) due to its amino acid-like structure and therefore bypasses the rate-limiting step of DA synthesis, the l-DOPA formation via tyrosine hydroxylase (TH) [5, 6]. It is converted into DA via aromatic l-amino acid decarboxylase (AADC) and restores DA availability in the brain [7].

Unfortunately, this kind of treatment only mitigates the symptoms, the process of neurodegeneration is not hindered [2, 8]. The cause of this pathogenesis is currently under intensive investigation, but not yet fully understood [9]. Two of the most discussed hypotheses are related to reactive oxygen species (ROS) [10] and mitochondrial dysfunction [9, 11]. But currently, there is no medication slowing the process of neurodegeneration, so treatment with l-DOPA still remains the gold standard and is combined with different co-treatments to optimize the pharmacokinetics of this drug [12]. A critical point in this context is the limited stability of l-DOPA: Besides enzymatic degradation, there are also spontaneous reactions by which l-DOPA as well as DA are bound to melanin [13], an auto-oxidation process during which ROS is formed [14].

In the past, several studies reported a cytotoxic effect of l-DOPA on neuronal cells in in vitro systems [15–17]. The cause for this toxicity has mainly been accounted to ROS formation during auto-oxidation [18]. In contrast, a few groups reported a neuroprotective effect of l-DOPA and a ROS scavenging function [19–23]. These contrary outcomes initiated in vivo studies and clinical trials to clarify these hypotheses. In comparison to in vitro experiments l-DOPA did not show neurodegenerative effects in vivo [24–27].

Although the physiological oxygen concentration in mammalian brains is low [28] and are hypoxic as compared to atmospheric oxygen levels, most if not all in vitro studies were performed under normoxic conditions and we suspected this as a plausible explanation for the observed differences. For this reason, we cultivated and treated neuronal cells in vitro with l-DOPA under normoxic and hypoxic conditions (2% O_2_) [28]. Due to the importance of mitochondrial dysfunction in PD patients we also focused especially on the central energy metabolism of these organelles.

## Results

### l-DOPA induced neurotoxicity is only of relevance under unphysiological normoxic conditions

**Fig. 1:**
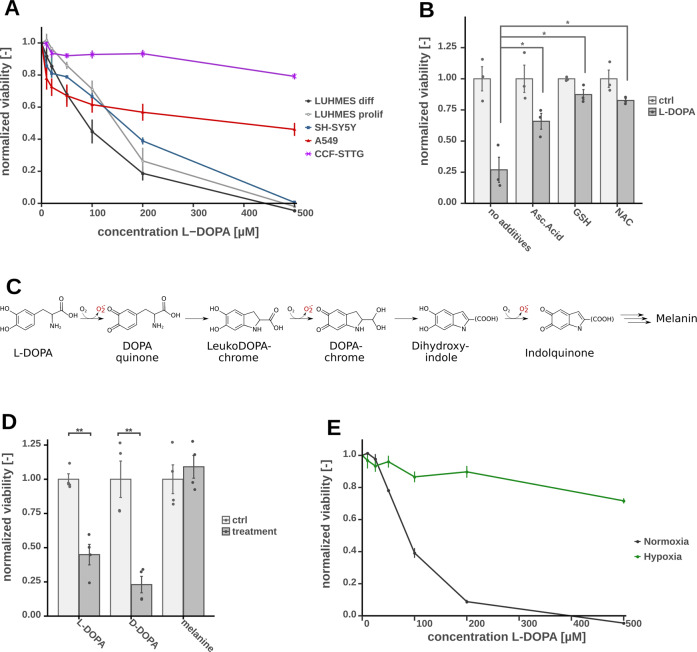
Viability assay based on resazurine after 24 h of l-DOPA treatment of LUHMES, displayed with the means and standard errors and normalized to the control group without l-DOPA, significance level were calculated using Welch’s *t*-test (*p-value < 0.05, **p-value < 0.01). **A** Viability of additional cell types treated with varying l-DOPA concentrations including fully differentiated (diff) LUHMES as well as proliferating (prolif) LUHMES (*n* = 4). **B** Co-incubation of with different anti-oxidant reagents GSH (1 mM), NAC (1 mM) or ascorbic acid (500 µM) together with 100 µM l-DOPA (*n* = 3). **C** Overview of the auto-oxidation process of l-DOPA including ROS formation. **D** Comparison of toxicity of l-DOPA (200 µM), d-DOPA (200 µM), and melanin formed from 200 µM l-DOPA solution (*n* = 3), **E** Comparison of cytotoxic effect of l-DOPA under normoxic and hypoxic conditions (*n* = 4).

To reveal molecular mechanisms of l-DOPA-mediated toxicity in in vitro systems, we compared the cytotoxicity of l-DOPA in a dose-dependent manner in human cell lines of different tissues. We determined l-DOPA mediated toxicity 24 h post treatment via a resazurine based viability assay (Fig. 1A) in neuronal (LUHMES), astrocytoma (CCF-STTGI), neuroblastoma (SH-SY5Y) and lung carcinoma (A549) cells. We observed that differentiated neuronal LUHMES cells (LUHMES diff) were most vulnerable to l-DOPA and that viability in this system decreased to 20–40% for 200 µM l-DOPA as compared to the control. Non-differentiated LUHMES as well as neuroblastoma cells SH-SY5Y were similar susceptible. Interestingly, both tested non-neuronal cells, A549 lung carcinoma and CCF-STTGI astrocytoma were much less vulnerable to l-DOPA indicating a higher sensitivity of neuronal cells. To validate if the observed cytotoxicity is mediated by reactive intermediates of l-DOPA (auto-) oxidation, we repeated the viability assays in the presence of antioxidant reagents such as GSH, NAC and ascorbic acid. Since all ROS scavengers attenuated cytotoxicity, we concluded that l-DOPA toxicity is to a significant extent caused by generated ROS or toxic intermediates and not exclusively by l-DOPA itself (Fig. 1B, C). To exclude toxicity mediated by enzymatic catalyzed l-DOPA reactions instead of auto-oxidation, we applied the DOPA enantiomer d-DOPA to the cells and measured cell viability and observed a comparable effect as for l-DOPA (Fig. 1D). Finally, we evaluated the impact of melanin on cell viability (Fig. 1D) and did not detect a significant toxicity of this end product of l-DOPA auto-oxidation. This is inline with our observation that already after 12 h all l-DOPA was auto-oxidized to melanin in a cell-free assay, while the viability is only decreasing for the first 12 h of l-DOPA treatment (Fig. S1a, b). Taken together, we demonstrated that non-enzymatic l-DOPA auto-oxidation products exhibited the highest toxicity in the tested neuronal cells.

The formation of ROS during l-DOPA auto-oxidation under normoxic conditions has been well described [29, 30], but the oxygen tension in neuronal cells under in vivo conditions is only 0.5–5% [28]. For this reason, we studied l-DOPA stability and toxicity under hypoxic conditions. We first tested the amount of l-DOPA auto-oxidation in a cell-free assay under hypoxia and could indeed confirm that degradation of l-DOPA was strongly reduced and <20% of l-DOPA was converted to melanin in a period of 12 h (Fig. S1c). We then determined cytotoxicity under hypoxic conditions in LUHMES cells and, as expected, cytotoxic effects were strongly decreased due to reduced oxygen tension (Fig. 1E, S1d). Based on these observations, we concluded that most of the previously described neurotoxic effects of l-DOPA can be at least partially attributed to artificially high levels of oxygen in in vitro experiments.

### Double-edged role of l-DOPA in intracellular ROS homeostasis

**Fig. 2:**
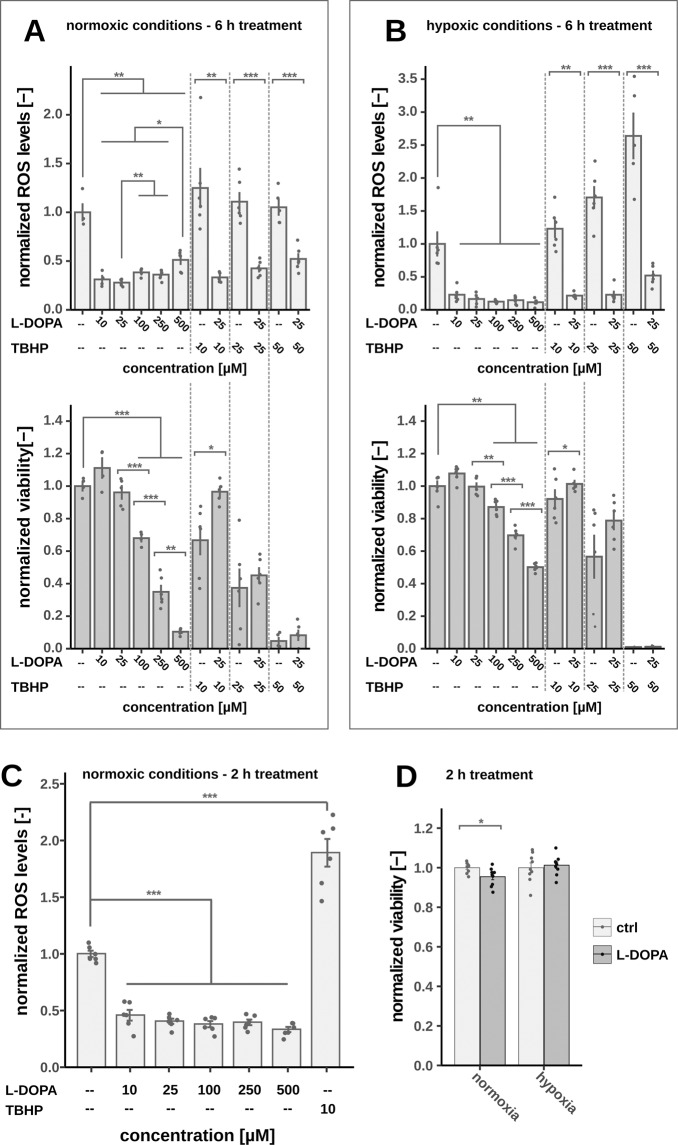
Depending on oxygen tension, L-DOPA either acts as ROS producer or ROS scavenger. Combined NR viability assay and intracellular ROS assay after 6 h of l-DOPA treatment under normoxic (**A**) and hypoxic (**B**) conditions including TBHP as a positive control for increased ROS formation and cytotoxicity (*n* = 5). **C** Intracellular ROS level after 2 h of l-DOPA treatment (*n* = 6). **D** Normalized viability based on NR after 2 h of l-DOPA treatment (200 µM) under normoxic and hypoxic conditions (*n* = 9). All experiments were done using fully differentiated LUHMES cells, mean, standard error and the single replicates are displayed, significance level were calculated using Welch’s *t*-test (*p-value < 0.05, **p-value < 0.01, ***p-value < 0.001).

Based on our findings that hypoxia limits auto-oxidation of l-DOPA, we were wondering if the catechol moiety of l-DOPA can even act as a ROS scavenger [22, 23]. Interestingly, l-DOPA treatment was able to reduce intracellular ROS down to 30% and to quench artificial ROS induced by TBHP treatment independent of the oxygen tension (Fig. 2A, B). Higher levels of TBHP induced enough ROS to almost completely kill the neuronal cells and intriguingly cell viability was partially restored when l-DOPA was supplemented (Fig. 2A, B). Even under normoxic conditions, l-DOPA was initially able to quench cellular ROS, but ROS levels increased in a dose-dependent manner after 6 h when l-DOPA auto-oxidation proceeded (Fig. 2A). This dose-dependent increase of ROS did not occur when incubating for only 2 h (Fig. 2C). The effect of l-DOPA on the cell viability was only minimal for this shortened treatment regardless of the oxygen levels (Fig. 2D). To exclude a secondary effect via l-DOPA-induced GSH dependent ROS scavenging, we measured intracellular GSH levels and determined gene expression of glutathione reductasse (GSR). Neither GSH level nor GSR expression was significantly decreased after L-DOPA treatment (Fig. S2). In summary, these findings indicate two opposing kinetics: l-DOPA itself had the capability to quench intracellular ROS, but l-DOPA auto-oxidation generated significant levels of cytotoxic ROS, in particular under artificial normoxic conditions.

### Impact of l-DOPA treatment on central energy metabolism

Based on these observations we were next interested to study auto-oxidation independent effects of l-DOPA on neuronal energy metabolism. We decided to study these effects in the neuronal LUHMES cell line, as these cells do not show measurable AADC activity and thus prevent DA production in our hands (Fig. S3). For this reason, experiments with these cells under hypoxic conditions combined with shorter treatment duration time disentangled metabolic effects of l-DOPA itself from auto-oxidation and DA mediated effects.

**Fig. 3:**
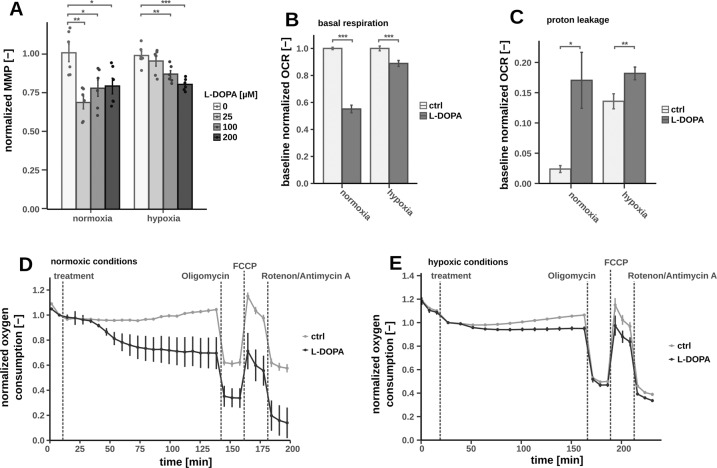
Mitochondrial membrane potential (MMP) and oxygen uptake under normoxic and hypoxic conditions after 2 h of l-DOPA treatment on fully differentiated LUHMES cells, significance level were calculated using Welch’s *t*-test (*p-value < 0.05, **p-value < 0.01, ***p-value < 0.001). **A** MMP with *n* = 6; **B** Calculated basal respiration after l-DOPA treatment (*n* = 13); **C** Calculated proton leak after l-DOPA treatment (normoxia: *n* = 4, hypoxia: *n* = 13); OCR over time under normoxic (**D**) or hypoxic conditions (**E**) after injections with l-DOPA, Oligomycine (Olig), FCCP and Rotenone/Antimycine A (Rot-AA) (**D**: *n* = 4; **E**: *n* = 5).

**Fig. 4:**
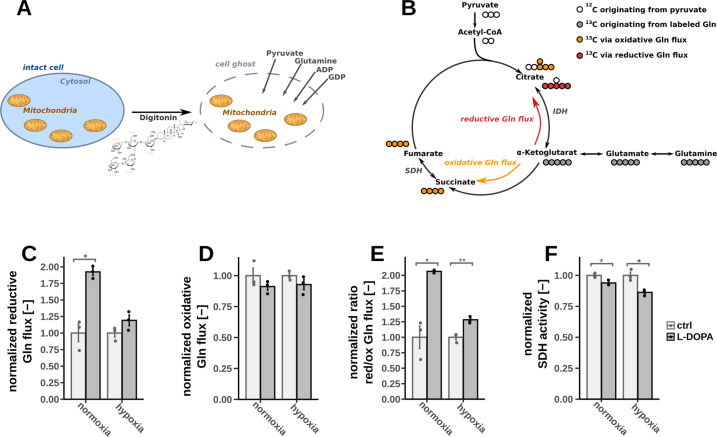
Multiple assays under after 2 h of l-DOPA treatment on fully differentiated LUHMES cells, significance level were calculated using Welch’s *t*-test (*p-value < 0.05, **p-value < 0.01, ***p-value < 0.001). **A** Intracellular ATP levels (*n* = 10). **B** Combined NAD and NADH level intracellular (normoxia: *n* = 4, hypoxia: *n* = 5). **C** Combined NADP and NADPH level intracellular (*n* = 6). **D** MMP after supplementation of NAD under hypoxic conditions (*n* = 6).

We first observed a strongly reduced mitochondrial membrane potential (MMP) (Fig. 3A), indicating overall impaired mitochondrial activity. One mechanism to attenuate mitochondrial function could be the miss-incorporation of l-DOPA instead of tyrosine into mitochondrial proteins [31, 32]. However, supplementation of high amounts of tyrosine did not rescue MMP in our experimental setup (Fig. S4) indicating a different mode of action. Impaired mitochondrial function was further confirmed by the measurement of reduced respiration. Both basal and maximum respiration (after mitochondrial membrane depolarization) were reduced (Fig. 3B, D, E). In addition, we found that mitochondrial proton leakage was increased (Fig. 3C, D, E). To obtain more detailed insights into mitochondrial metabolism, we selectively permeabilized the cytosolic membrane with digitonin and applied stable-isotope tracing. This setup allowed us to profile mitochondrial metabolism independently of the residual cellular context (Fig. 4A) [33]. We applied uniformly labeled ^13^C_5_-glutamine to determine oxidative and reductive mitochondrial TCA cycle fluxes. Oxidative glutamine metabolism (glutaminolysis) produces M4-citrate isotopologues whereas M5 citrate is generated via the reductive path (Fig. 4B). We observed a significant increase of reductive TCA activity as compared to the oxidative flux upon l-DOPA treatment (Fig. 4C, D, E). Moreover, based on the ratio of M4 fumarate to M4 succinate, we found that succinate dehydrogenase (SDH) activity was significantly reduced as well (Fig. 4F). Taking all these results together, l-DOPA attenuated MMP, by inhibiting respiration and potentially by inducing a proton leak. As a consequence NADH could not be oxidized to NAD+ by complex I and increased levels of NADH were compensated by reductive carboxylation through isocitrate dehydrogenase (IDH).

**Fig. 5:**
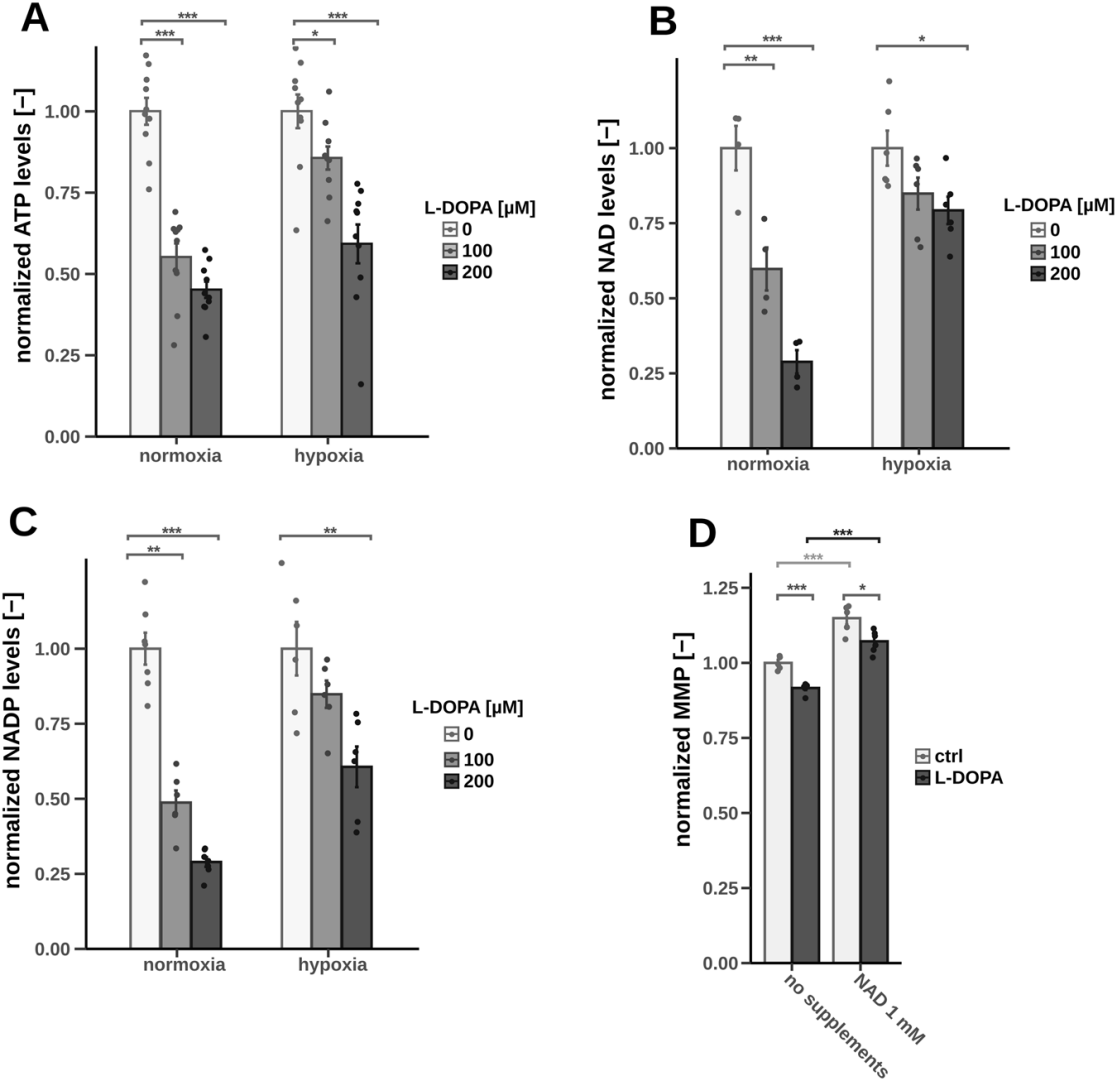
Combined approach of selective permeabilization and stable isotope labeling after 2 h of l-DOPA treatment (200 µM) on fully differentiated LUHMES cells under normoxic and hypoxic conditions (significance levels were calculated using Welch’s *t*-test (*p-value < 0.05, **p-value < 0.01), *n* = 3). **A** Schematic overview of the selective permeabilization via digitonin generating cell ghosts with intact mitochondria. **B** Schematic overview of the carbon transitions using the substrates ^13^C_5_-glutamine and ^12^C-pyruvate. **C** Reductive Gln flux calculated via the ratio of M5 citrate to M5 α-ketoglutarate. **D** Oxidative Gln flux calculated via the ratio of M4 citrate to M5 α-ketoglutarate. **E** Ratio between reductive and oxidative Gln flux calculated via the ratio of M5 citrate to M4 citrate. **F** SDH activity calculated via the ratio of M4 fumarate to M4 succinate.

Next, we measured intracellular ATP levels to investigate whether the l-DOPA-induced mitochondrial dysfunction results in a decreased cellular energy load. We observed that ATP levels were indeed reduced by l-DOPA in a dose-dependent manner (Fig. 5A). Interestingly, we additionally observed that the combined pool of NAD+/NADH was depleted as well (Fig. 5B) and observed a similar trend for NADP+/NADPH (Fig. 5C). Supplementation of additional NAD+showed a positive effect on the MMP in general and was able to counteract the impairing effect of l-DOPA (Fig. 5D). But interestingly the supplementation was not able to abolish the effect of l-DOPA indicating that NAD+/NADH depletion is not the sole reason behind l-DOPA-induced mitochondrial impairment. We were also wondering if the observed co-factor depletion might be elicited from PARP-dependent DNA repair mechanisms [34]. We observed overall less DNA damage in cells treated with l-DOPA (Fig S5a). But co-incubation with the PARP inhibitor olaparib [35] did not restore NAD+/NADH levels questioning the participation of PARP-dependent repair mechanisms and instead of indicating that reduced DNA damage might be a consequence of decreased intracellular ROS levels.

### Transcriptomics analysis revealed further alterations induced by l-DOPA

To gain more profound insights into the l-DOPA mediated effects, we performed a gene expression analysis by RNA sequencing technology. We found that the genes encoding NADH synthesis and depletion remained mainly unaffected (Fig. S6a). Only one of these genes encoding sirtuin 7 (SIRT7) was significantly higher expressed (Fig. S6b). SIRT7 interacts with nuclear respiratory factor 1 (NRF1) and therefore is known to directly impair mitochondria [36]. To test the impact of the deacetylating activity of SIRT7 in this experimental setup, we employed siRNA-mediated gene knockdown (KD). However, SIRT7 KD was not able to revert the MMP impairment refuting the involvement of SIRT7 in l-DOPA mediated mitochondrial depolarization (Fig. S6c, d).

**Fig. 6:**
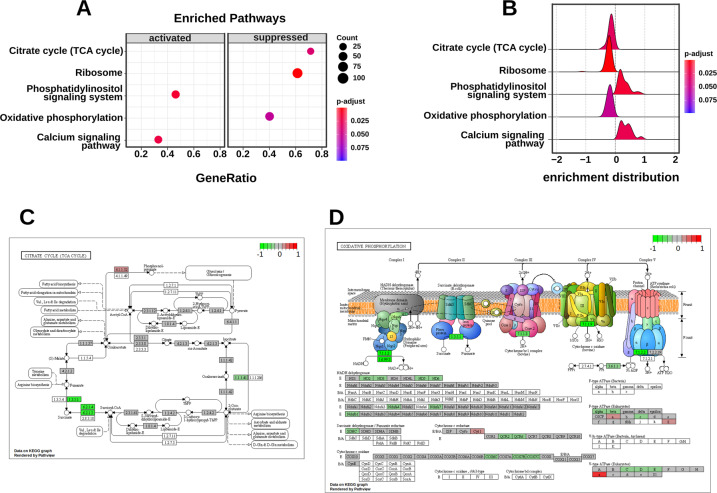
Overview of the results of the GSEA analysis. **A** Enrichment plot displaying activated and suppressed pathways including the count of annotated genes, the adjusted *p*-value, and the ratio of activated or suppressed genes. **B** Rigde plot displaying the enrichment distribution of the pathways over the log2 foldchange. **C** Overview of the normalized downregulation (green) and upregulation (red) of the genes of the TCA cycle. **D** Overview of the normalized downregulation (green) and upregulation (red) of the genes of oxidative phosphorylation.

Finally, we performed a Gene Set Enrichment Analysis (GSEA) to profile global gene expression including pathways of central energy metabolism (Fig. 6A, B). l-DOPA generally tended to significantly lower expression of TCA cycle-related genes (Fig. 6A–C) and oxidative phosphorylation, especially SDH, even though the difference for the oxidative respiration was not significant (*p*-value 0.069, Fig. 6A, B, D). Transcription of ribosomal RNA was also inhibited which was most likely linked to general impaired energy metabolism (Fig. S7) [37, 38]. Moreover, the calcium (Ca^2+^) dependent signaling pathway seemed to be strongly impacted (Fig. S8). The phosphatidylinositol signaling pathway, which is known to play a central role in Ca^2+^ signaling, was also higher induced (Fig. S9).

## Discussion

Independent of its function as a precursor for DA synthesis, we demonstrated that l-DOPA can mediate both, a neurotoxic and a neuroprotective activity. Interestingly, the precise kind of action depends directly on the oxygen tension. Under normoxic oxygen levels, auto-oxidation of l-DOPA and linked ROS production prevails, while under hypoxic conditions l-DOPA auto-oxidation is heavily reduced. With its catechol moiety, l-DOPA even acts as an antioxidant. With our results, we can explain the discrepancy between the toxicity observed in previous in vitro experiments and opposing results in in vivo studies [39]. All in vitro experiments, that we are aware of, had been performed under normoxic conditions and thus ROS-mediated cytotoxicity caused by l-DOPA auto-oxidation prevailed and this was confirmed by this study. However, the level of oxygen in the brain in vivo has been estimated to be below 5%. In our current study we demonstrated that under these conditions l-DOPA is stabilized, the neurotoxic auto-oxidation is drastically decelerated. Most likely, this is part of the reason why the severe cytotoxic effects of l-DOPA observe impairment of neuronal mitochondrial function by l-DOPA in the absence of oxygen-dependent auto-oxidation and oxidative cell damaged in various in vitro experiments could not be confirmed in in vivo models.

We demonstrated that during the initial phase of l-DOPA treatment, even under normoxia, the ROS scavenging action had dominated and was then oxygen, time, and dose dependent more and more replaced by cytotoxic ROS production due to l-DOPA auto-oxidation. This dual mode of l-DOPA action might be the reason why previous in vitro studies described both, neurotoxic and neuroprotective effects against extracellular ROS [19–21]. In contrast to the study of Han et al. [21] we did not observe increased GSH levels, but GSH was even slightly reduced which is a known potential effect of l-DOPA itself [40]. Instead of activating the ROS defense mechanisms [21], the molecule l-DOPA itself acts as a ROS scavenger during the shortened treatment duration. Similar observations were already published by other groups strengthening this conclusion [22, 23]. So overall our data suggest an oxygen-dependent dual effect of l-DOPA: Under normoxic conditions l-DOPA auto-oxidation produces cytotoxic ROS, whereas under hypoxia and during shorter treatment duration l-DOPA is more stable and the ROS scavenging capacity prevails. Moreover, we observed that neuronal cell lines were much more vulnerable than other human cell types to l-DOPA-mediated ROS. Neuronal cells are known to have only limited capacities for ROS defense mechanisms and require interactions with other cell types harboring the neuron-astrocyte-glutathione shuttle [41].

With respect to the central energy metabolism, we observed that l-DOPA directly attenuated genes encoding for enzymes of oxidative phosphorylation including SDH and complex I. Based on ^13^C metabolic flux experiments, we successfully confirmed these observations. The metabolic flux through SDH was decreased along with an increase of reductive glutamine metabolism [42]. Furthermore, l-DOPA treatment reduced the MMP as well as intracellular ATP levels. In line with all these observations, the overall respiration of the cells was decreased while the proton leakage of the mitochondria were slightly increased, all indicative of impaired mitochondrial function. These results may also put a new perspective on previous in vivo studies that reported similar results [43]. But these observations were mainly interpreted by l-DOPA-derived DA – DA itself is known to impair the central energy metabolism as well [18, 44]. Our study suggests that the effects of l-DOPA itself might be a part of these previous in vivo results.

Even more interesting there is also a link to PD itself. Many risk factors for PD such as mutations in *Parkin*, *PINK1* or *LRRK2* are linked to mitophagy, reduced MMP, and eventually impaired ATP production [45–48]. This is in agreement with observations by Hattingen *et al*. who showed that PD patients in general have lower ATP levels in the brain as compared to healthy individuals [49]. However, there are currently no clinical magnetic resonance spectroscopy (MRS) studies that examined the direct and time-resolved effect of l-DOPA treatment in particular within the brain of PD patients. There are already studies to test drugs like ursocholanic acid on their beneficial effect in restoring the MMP and ATP levels in the brain as a treatment for PD [50]. These kinds of treatments might be even more important if our findings would be confirmed in vivo. Besides the lowered MMP and ATP levels, we also observed lower levels of combined NAD+ and NADH as well as NADP+ and NADPH after l-DOPA treatment. There are already approaches that test rescuing effects of NAD+ precursors against neurotoxicity [51–53]. Direct supplementation of NAD+ confirmed a positive effect in our study as well.

Another important link between the affected ATP level and PD might be the influence on Ca^2+^ homeostasis. There is lots of evidence that Ca^2+^ is another crucial aspect in PD pathogenesis [54–57], resulting in multiple clinical trails [58]. In our transcriptomics analysis we observed a significant enrichment of the calcium-dependent signaling pathway. Ca^2+^ is essential for mitochondrial ATP synthesis due to the Ca^2+^ dependent activation of the electron transport chain, the Ca^2+^-ATPase as well as the overall impact on the NAD+/NADH homeostasis and the MMP [59, 60]. Previous studies already showed the potential impact of l-DOPA treatment in the context of Ca^2+^ homeostasis [61, 62], other groups even reported about direct connections between Ca^2+^ signaling and l-DOPA-induced dyskinesia (LID) [63, 64].

## Conclusion

While it is widely accepted that l-DOPA induced neurodegeneration is artificial to in vitro studies, we confirmed that the increased oxygen tension as compared to physiological levels in common cell culture setups seemed to be the origin for this discrepancy between in vitro and in vivo studies. We eliminated the artificial ROS formation and were even able to confirm a ROS scavenging effect of l-DOPA itself. Interestingly there are various links to previously published in vivo studies strengthening the assumption that the hypoxic in vitro setup better represents in vivo conditions compared to normoxia in vitro studies. The observed alterations of the central energy metabolism might be highly important for clinical approaches. Even though there have been several clinical studies to distinguish potential negative effects of l-DOPA in PD patients in the past [65, 66], specific investigation of mitochondrial function upon l-DOPA treatment might be essential to confirm our study in a clinical matter. This applies especially for familial PD patients with already altered mitochondrial function. Overall our study might be a crucial factor for predicting LID and might be one of the missing pieces in the puzzle of optimizing and personalizing PD treatment.

## Materials and methods

### Cell culture

If not stated otherwise we used Lund human mesencephalic (LUHMES) cells, an immortalized mesencephalon cell line, which can be differentiated to post-mitotic cells [67, 68]. For LUHMES cultivation flasks or well plates were coated with 50 µg/ml Poly-L-Ornithin (Sigma-Aldrich, P3655) and 1 µg/ml fibronectin (Sigma-Aldrich, F1141). LUHMES cells were cultured in Advanced DMEM/F-12 medium (Gibco, 12634010) supplemented with 2 mM glutamine (Sigma-Aldrich, G7513), 1x N2 supplement (Gibco, 17502048) and 40 ng/ml FGF (R&D Systems, 4114-TC). For differentiation the proliferation medium was replaced with differentiation medium without FGF but 1 mM db-cAMP (Sigma-Aldrich, D0627), 1 µg/ml tetracyclin (Sigma-Aldrich, T7760) and 20 ng/ml GDNF (R&D Systems, 212-GD). After two days the cells were seeded with a density of 14 × 10^4^ cells per cm^2^. After additional 3 days the differentiation were complete and the treatments started. SH-SY5Y and A549 cells were cultivated in DMEM high glucose medium (Gibco, 41965039) supplemented with 10 % FBS (Gibco, A3840401) and CCF-STTGI were cultivated in RPMI 1640 (Gibco, 21875034) supplemented with 10% FBS. All proliferating cells were seeded the day before the experiment with a density of 7 × 10^4^ cells per cm^2^. Cells were treated for different durations between 2 and 24 h with freshly prepared solutions in a complete medium including l-DOPA (Sigma-Aldrich, D9628), d-DOPA (Sigma-Aldrich, D9378), ascorbic acid (Sigma-Aldrich, A4403), glutathione (GSH; Sigma-Aldrich, G4251), N-Acetylcysteine (NAC; Sigma-Aldrich, A9165), tert-butyl hydroperoxide (TBHP; Sigma-Aldrich, 458139) and β-Nicotinamide adenine dinucleotide (NAD, Sigma-Aldrich N3014). Melanin obtained by incubating l-DOPA in a complete growth medium for 24 h at 37 °C. If experiments were done under hypoxic conditions, the cells were transferred 2 days prior to the start of the treatment to the hypoxia chamber containing 2% oxygen which represents the oxygen tension in the mammalian mid brain [28].

### Viability measurements

Cell viability for experiments over 24 h were measured using resazurine fluorescence assay [69]. Resazurine (Sigma-Aldrich, R7017) was added to the cells to a final concentration of 5 µg/ml and incubated for 1 h at 37 °C. Viability was measured at excitation 555 nm and emission 595 nm. When the cells were treated for only 2–6 h the viability was measured using the neutral red (NR) assay [70]. NR (Sigma-Aldrich, N2889) was added to the cells to a final concentration of 10 µg/ml and incubated for 2 h at 37 °C. The medium was discarded and cells were washed once using phosphate-buffered saline solution (PBS). Afterwards the cells were lysated using a solution with 50% ethanol (EtOH; HPLC grade), 49% water and 1% acetic acid (Sigma-Aldrich, 695092) and incubated for 10 min before measuring the fluorescence at excitation at 530 nm and emission at 645 nm. The resazurine assay was used for the long-term experiments because of its lack of washing steps which could wash cells away and falsify the viability results. The NR assay was used for the short-term treatments because of its compatibility with the ROS assay and the independence from the NAD^+^/NADH ratio inside the cells. The different assays were directly compared for 24 h treatment and showed the same trend (data not shown).

### Intracellular ROS measurements

The intracellular ROS measurements was performed using the fluorescence dye 2,7-dichlorfluoresceine (DCFDA; Sigma-Aldrich D6883) [71]. The cells were seeded in black fluorescence microplates. The cells were incubated with 25 µM DCFDA for 45 min at 37 °C. Afterwards the medium was discarded, the cells were washed once with PBS and the treatment with l-DOPA or TBHP for 2–6 h was started. Intracellular ROS levels were measured with excitation at 485 nm and emission at 535 nm. If a NR assay was added to the experiment, 2× NR stock solution was added (same volume as already in the well) after the ROS measurement and the assay was performed as described above.

### MMP measurements

The mitochondria membrane potential (MMP) measurement is based on the fluorescence dye JC-10 and was obtained from Sigma-Aldrich (MAK159). The cells were seeded in black fluorescence 96 well plates and treated with 50 µl medium containing various supplements. After 2 h 25 µl of buffer A containing JC-10 were added. After additional 45 min 25 µl of buffer B was added. The fluorescence measurements were done at excitation 540 nm and emission 590 nm as well as excitation at 490 nm and emission at 530 nm. The ratio between the fluorescence signals excited at 540 nm/490 nm represents the MMP.

### ATP measurements

The adenosine triphosphate (ATP) measurement is based on luminescence and was purchased from Abcam (ab113849). The assay was done according to the manufacturer’s instructions. The cells were treated in 50 µl medium in a white 96 well luminescence plate. After the treatment 25 µl detergent solution were added and vortexed for 5 min. Afterwards 25 µl substrate solution were added and again vortexed for 5 min. After 10 min of incubation in the dark, the intracellular ATP levels were measured via luminescence.

### NAD(P) measurements

The NAD+ and NADP+ measurements are based on luminescence and were purchased from Promega (G9071, G9081). The reaction mixtures were prepared according to the manufacturer’s instructions. The cells were treated in 50 µl medium in a white 96 well luminescence plate. After the treatment 50 µl assay buffer were added and the mixture was incubated for 90 min before measuring the luminescence signal.

### Oxygen uptake

To measure the oxygen uptake the Agilent Seahorse XE96 system was used (Wave software 2.4.2). Due to the prolonged time of the assay and the vulnerability of the cell line we prepared a specific Seahorse medium in house instead of using the standard Agilent Seahorse medium. We completely reformulated the Advanced DMEM/F12 medium using DMEM 5030 (Sigma-Aldrich, D5648), F12 nutrition mix (Gibco, 15172529), and insulin-transferin-selenium-ethanolamine supplement (Gibco, 51500056). We weighed in all missing compounds simultaneously, obtaining all chemicals except AlbuMAX II (Gibco, 11560376) from Sigma-Aldrich. We replaced the usual bicarbonate buffer by HEPES (15 mM) to guarantee a stable pH. For the experiments we seeded 3 × 10^4^ cells per well. One day before the differentiations were finished we replaced the normal differentiation medium with our self-made Seahorse differentiation medium and finished differentiation in a carbon-dioxide-free incubator. Besides the 2 h treatment using l-DOPA a normal Agilent MitoStress test (Agilent, 103015) was used including oligomycin to inhibit complex V (1 µM), carbonyl cyanide-p-trifluoromethoxyphenylhydrazone (FCCP) to uncouple oxygen consumption from ATP production (0.5 µM for normoxia, 1 µM for hypoxia) and rotenone and antimycin A to inhibit complex I and III (each 0.5 µM). Under hypoxic conditions, wells containing NaSO_3_ (Sigma-Aldrich, S0505) were added as recommended by the manufacturer. For the Seahorse experiments the oxygen concentration were increased from 2 to 3% to guarantee the proper function of the instrument.

### Mitochondrial metabolic flux measurements

In general, we used the same experimental setup as previously described [33]. After the 2 h l-DOPA treatment, the medium was discarded and the cells were treated with 50 µg/ml digitonin (Sigma-Aldrich, D141) for 2 min at 37 °C to selectively perforate the cell membrane leaving the mitochondria membrane intact. Afterwards the cells were incubated for 30 min at 37 °C in the assay buffer containing 1 mM pyruvate, 1 mM ^13^C_5_-glutamine (Sigma-Aldrich, 605166), 2 mM ADP (Sigma-Aldrich, A5285), 0.5 mM GDP (Sigma-Aldrich, G7127), and 200 µM l-DOPA. After the treatment, the cell ghosts were washed with 0.9% NaCl and extracted as previously described [72]. Because of the two-phase extraction using methanol, water, and chloroform, it was possible to obtain the polar phase containing small polar metabolites as well as an interphase containing proteins, DNA and RNA. The polar phase was dried and measured via gas chromatography and mass spectrometry as previously described [73]. The raw data were analyzed using *MetaboliteDetector* [74].

### RNA purification and RNA sequencing

RNA samples were obtained using the same two-phase extraction as mentioned above, but on whole cells. RNA purification were done using the Machery-Nagel Nucleospin kit and followed the manufacturers instruction. The quality and integrity of total RNA was controlled on Agilent Technologies 2100 Bioanalyzer (Agilent Technologies; Waldbronn, Germany). The RNA sequencing library was generated from 10 ng total RNA using NEBNext Single Cell/Low Input RNA Library Prep Kit (New England BioLabs) according to the manufacture´s protocols. The libraries were sequenced on Illumina NovaSeq 6000 using NovaSeq 6000 S1 Reagent Kit (100 cycles, paired end run) with an average of 3 × 10^7^ reads per RNA sample. Primary analysis were done using the webtool *Galaxy* including *fastq trimming*, genome alignment and gene annotations (reference genome *human hg38*) as well as read counting, normalization and statistics using the methods *HISAT2*, *featureCounts*, and *edgeR* with a filter of at least 3 counts per million [75–78].

### Data analysis and visualization

Any further data analysis were done using Microsoft Excel and R. Treatment results were normalized by setting values of untreated replicates as 100%. Data are monitored with the mean and s.e.m if not stated otherwise. Data visualization were done using various R packages including *ggplot2*, *enrichplot*, *DOSE*, and *pathview* [79–81].

## Supplementary information

Supplementary methods

Supplementary figure legends

Supplemented Figure 1

Supplemented Figure 2

Supplemented Figure 3

Supplemented Figure 4

Supplemented Figure 5

Supplemented Figure 6

Supplemented Figure 7

Supplemented Figure 8

Supplemented Figure 9
