# Supplementary material for: Impairment of neuronal mitochondrial function by l-DOPA in the absence of oxygen-dependent auto-oxidation and oxidative cell damage
Source: Cell Death Discov. 2021 Jun 28;7:151. doi: 10.1038/s41420-021-00547-4 (PMC8257685; doi:10.1038/s41420-021-00547-4)
Supplement: Supplementary file 1 — Supplementary methods [file 41420_2021_547_MOESM1_ESM.docx]

**Impairment of neuronal mitochondrial function by L-DOPA in the absence of oxygen-dependent auto-oxidation and oxidative cell damage**

**Supplemented Material and Methods**

1.1 Melanine and L-DOPA measurements

Melanine quantification were done measuring the absorbance at 405 nm in a cell free experiment. L‑DOPA extractions from the medium and GC-MS measurements were performed as previously described^1^⁠.

1.2 GSH extraction and GSR transcription

Cells were incubated in a 12-well plate, treated as described in the main article, washed using 0.9 % NaCl solution and transfered on a cooling plate. Afterwards 100 µl water including 20 µg/ml internal standard (^13^C_2_^15^N_1_-GSH) and 100 µl 5 % tri-chloro acetic acid were added. Cells were scraped, transferred into reaction tubes, vortexed for 10 min at 1400 rpm and 4°C and centrifuged afterwards for 5 min at 17,000x g and 5 min. 150 µl of the supernatant were transferred into a LC-MS vial and measured immediately or stored at -80°C until measurement. Measurement were done using LC-MS as previously described.^2^⁠ GSR mRNA quantification was performed using the *edgeR* data obtained from the RNA sequencing data set described in the main article.

1.3 LUHMES AADC overexpression, western blot, qPCR and intracellular DA measurement

The generation of LUHMES overexpressing AADC (AADCox) including all following experiments were performed as described by *Delcambre*.^3^⁠

1.4 DNA damage

The DNA damage was quantified using the HCS CNA Damage kit obtained from Invitrogen (H10292). The assay was performed as described in the kit’s manual.

1.5 SIRT7 knockdown

The chemical transfection were performed using Lipofectamine RNAiMAX from Thermo Fisher (13778150). 30 pmol siRNA (Horizon Discovery, L-007774-01-0005) and 2 µl lipofectamine were dissolved in 150 µl Opti-MEM medium (Thermo Fisher, 31985070) and incubated for 20 min. The cells were seeded normally in 850 µl and prepared 150 µl Opti-MEM medium were added 3 days prior the experiments. The cell extraction was performed as described in the main article, the interphase was washed with MeOH and afterwards the RNA extraction was performed using the kit NucleoSpin RNA from Machery-Nagel. The qPCR afterwards was performed as previously described.^4^

1. Krämer, L., Jäger, C., Trezzi, J. P., Jacobs, D. M. & Hiller, K. Quantification of stable isotope traces close to natural enrichment in human plasma metabolites using gas chromatography-mass spectrometry. *Metabolites* **8**, (2018).

2. Meiser, J. *et al.* Loss of DJ-impairs antioxidant response by altered glutamine and serine metabolism. *Neurobiol. Dis.* **89**, 112–125 (2016).

3. Delcambre, S. In Vitro Metabolic Studies of Dopamine Synthesis and the Toxicity of L-DOPA in Human Cells. (University of Luxembourg, 2016).

4. Battello, N. *et al.* The role of HIF-1 in oncostatin M-dependent metabolic reprogramming of hepatic cells. *Cancer Metab.* **4**, 1–14 (2016).
