# Supplementary material for: Impairment of neuronal mitochondrial function by l-DOPA in the absence of oxygen-dependent auto-oxidation and oxidative cell damage
Source: Cell Death Discov. 2021 Jun 28;7:151. doi: 10.1038/s41420-021-00547-4 (PMC8257685; doi:10.1038/s41420-021-00547-4)
Supplement: Supplementary file 2 — Supplementary figure legends [file 41420_2021_547_MOESM2_ESM.docx]

**Impairment of neuronal mitochondrial function by L-DOPA in the absence of oxygen-dependent auto-oxidation and oxidative cell damage**

**Supplementary figure legends**

Fig S1. (A) L-DOPA degradation and melanin formation under normoxic conditions in pure medium without cells (L‑DOPA: n = 3; melanin: n = 6). (B) Viability assay based on NR with different points of time of L‑DOPA treatment (200 µM) resulting in time resolved killcurve for LUHMES cells under normoxic conditions (n = 6). (C) L‑DOPA degradation and melanin formation under hypoxic conditions in pure medium without cells (L‑DOPA: n = 3; melanine: n = 6; melanin: n = 6). (D) Viability assay based on NR with different points of time of L‑DOPA treatment (200 µM) resulting in time resolved killcurve for LUHMES cells under hypoxic conditions (n = 6).

Fig S2. (A) Intracellular GSH levels in LUHMES after 2 h L-DOPA (200 µM) treatment (n = 3). (B) GSR expression in LUHMES under hypoxic conditions after 2 h L‑DOPA treatment (200 µM) (n = 3). Significance levels were calculated using Welch’s t-test.

Fig S3. Gene expression (A) and western blot (B) of AADC in LUHMES wildtype (WT) and overexpressing AADC (AADCox). (C): intracellular DA levels of LUHMES WT and AADCox after 2.5 h L-DOPA treatment (50 µM) under normoxic conditions; DA levels for WT and AADCox without L-DOPA were below the limit of detection. n = 3.

Fig S4. MMP assay with 200 µM L‑DOPA and supplementation of additional tyrosine to the medium under normoxic conditions (n = 6). Significance levels were calculated using Welch’s t‑test.

Fig S5. (A) DNA damage kit after LUHMES were treated with L-DOPA or valinomycine as a positive control. Blue: Hoechst dye quantifying the total cell number indicating the toxicity of the treatment; Red: Alexa Fluor dye quantifying DNA damage. Treatment were 2 h of incubation with 200 µM L-DOPA or 30 µM valinomycine (n = 6). (B) Intracellular NAD levels after L-DOPA treatment with and without PARP inhibitor olaparib (n = 8). Significance levels were calculated using Welch’s t-test.

Fig S6. (A) Overview of the normalized downregulation (green) and upregulation (red) of the genes of the NAD metabolism after 2 h L-DOPA treatment (200 µM) under hypoxic conditions. (B) Normalized expression of SIRT7 after 2 h L-DOPA treatment (200 µM) under hypoxic conditions (n = 3). (C) Normalized expression of SIRT7 after KD using siRNA measured via qPCR (n = 6). (D) Normalized MMP after 2 h L‑DOPA treatment under hypoxic conditions for SIRT7 KD and siRNA ctrl (n = 5). Significance levels were calculated using Welch’s t-test.

Fig S7. Overview of the normalized downregulation (green) and upregulation (red) of the genes of the ribosome transcription

Fig S8. Overview of the normalized downregulation (green) and upregulation (red) of the genes of the calcium signaling pathway

Fig S9. Overview of the normalized downregulation (green) and upregulation (red) of the genes of the phosphatidylinositol signaling system
