# Supplementary figures and images for: Impairment of neuronal mitochondrial function by l-DOPA in the absence of oxygen-dependent auto-oxidation and oxidative cell damage
Source: Cell Death Discov. 2021 Jun 28;7:151. doi: 10.1038/s41420-021-00547-4 (PMC8257685; doi:10.1038/s41420-021-00547-4)

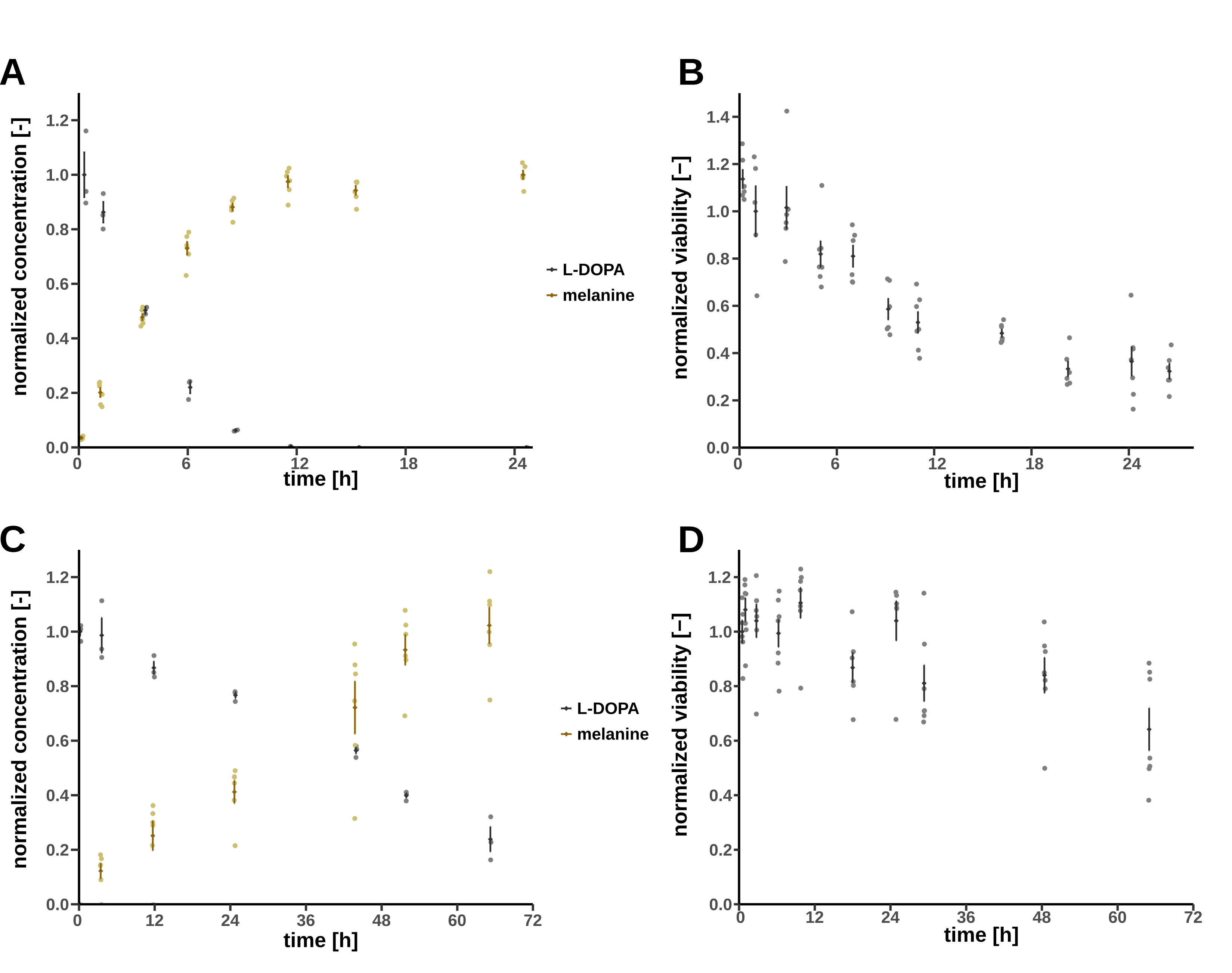

Supplement: Supplementary file 3 — Supplemented Figure 1 [file 41420_2021_547_MOESM3_ESM.png]

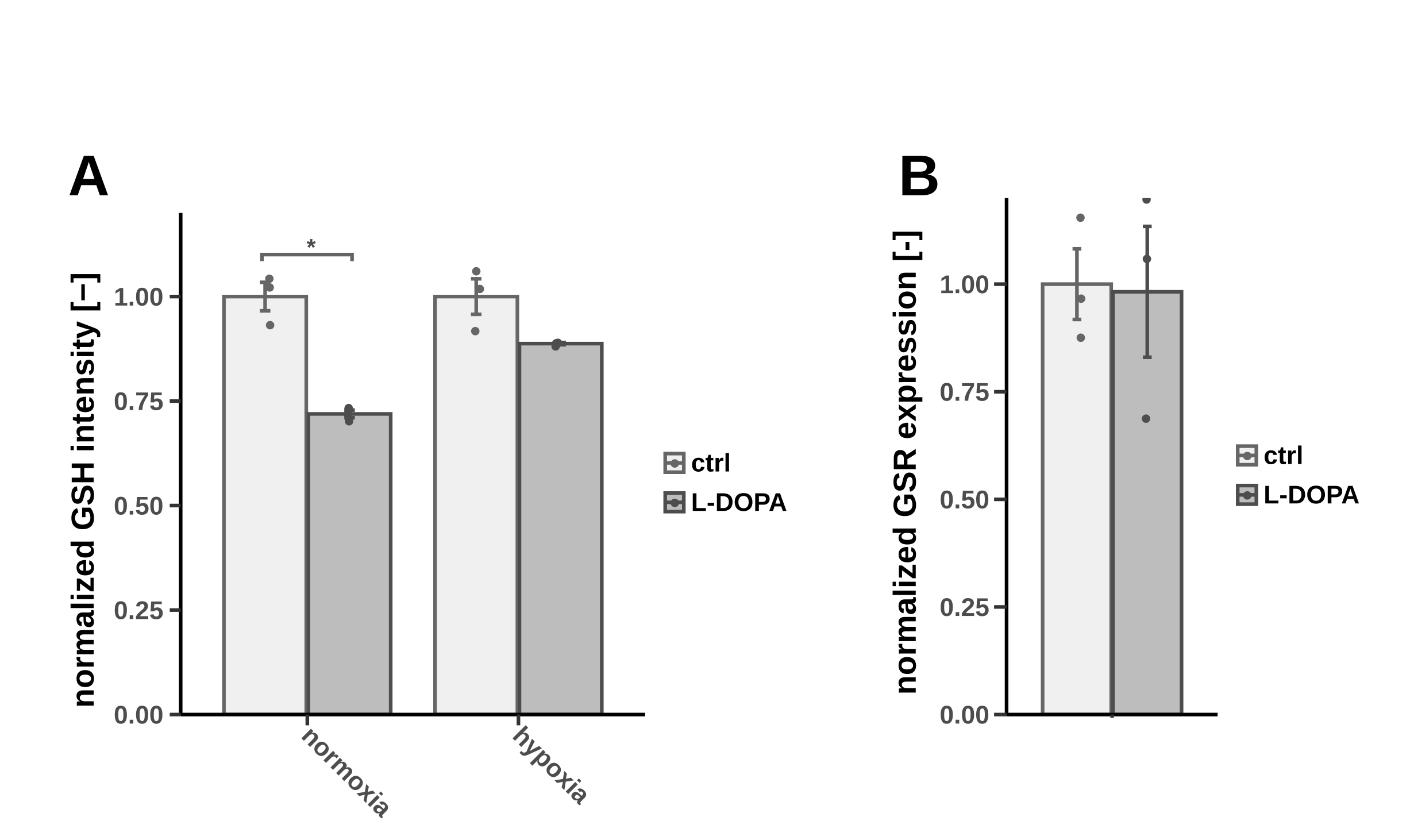

Supplement: Supplementary file 4 — Supplemented Figure 2 [file 41420_2021_547_MOESM4_ESM.png]

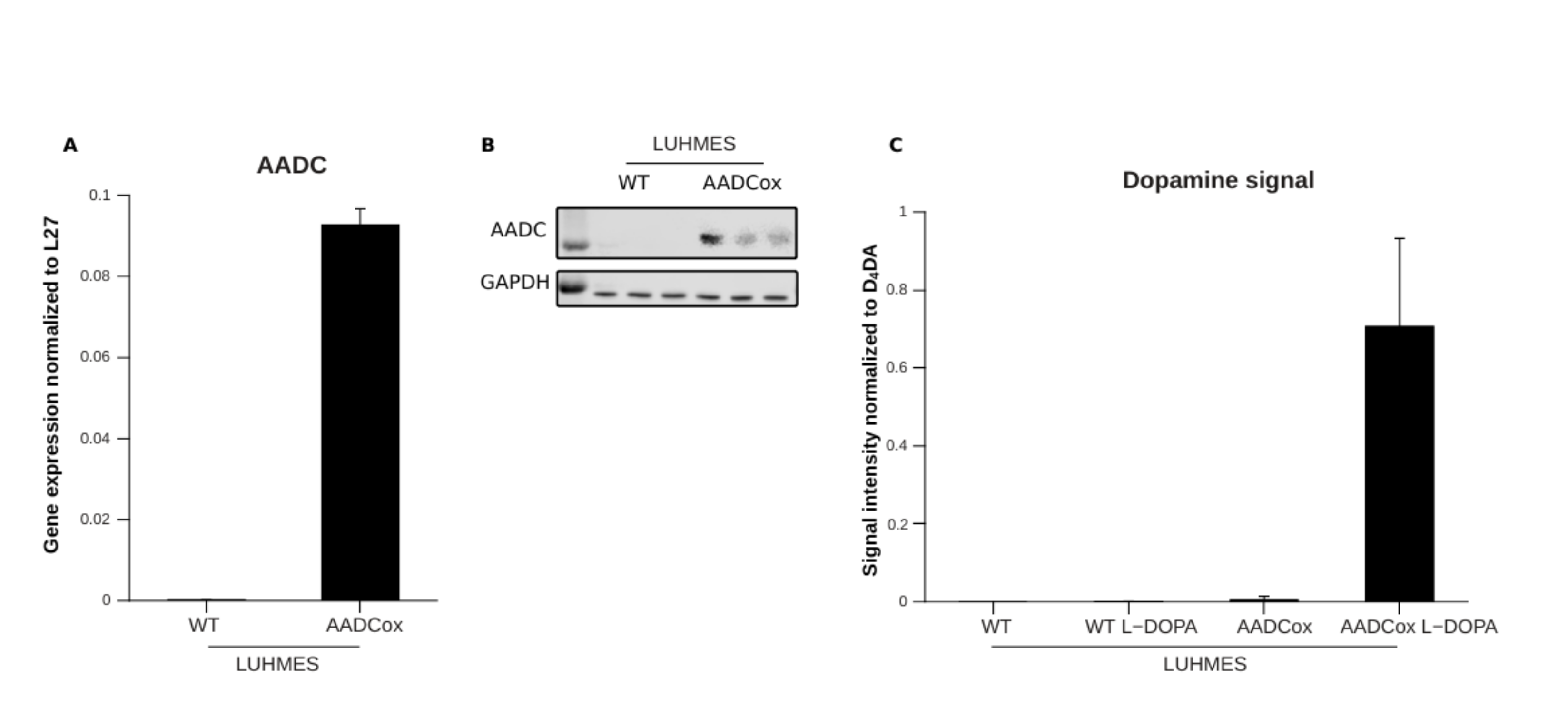

Supplement: Supplementary file 5 — Supplemented Figure 3 [file 41420_2021_547_MOESM5_ESM.png]

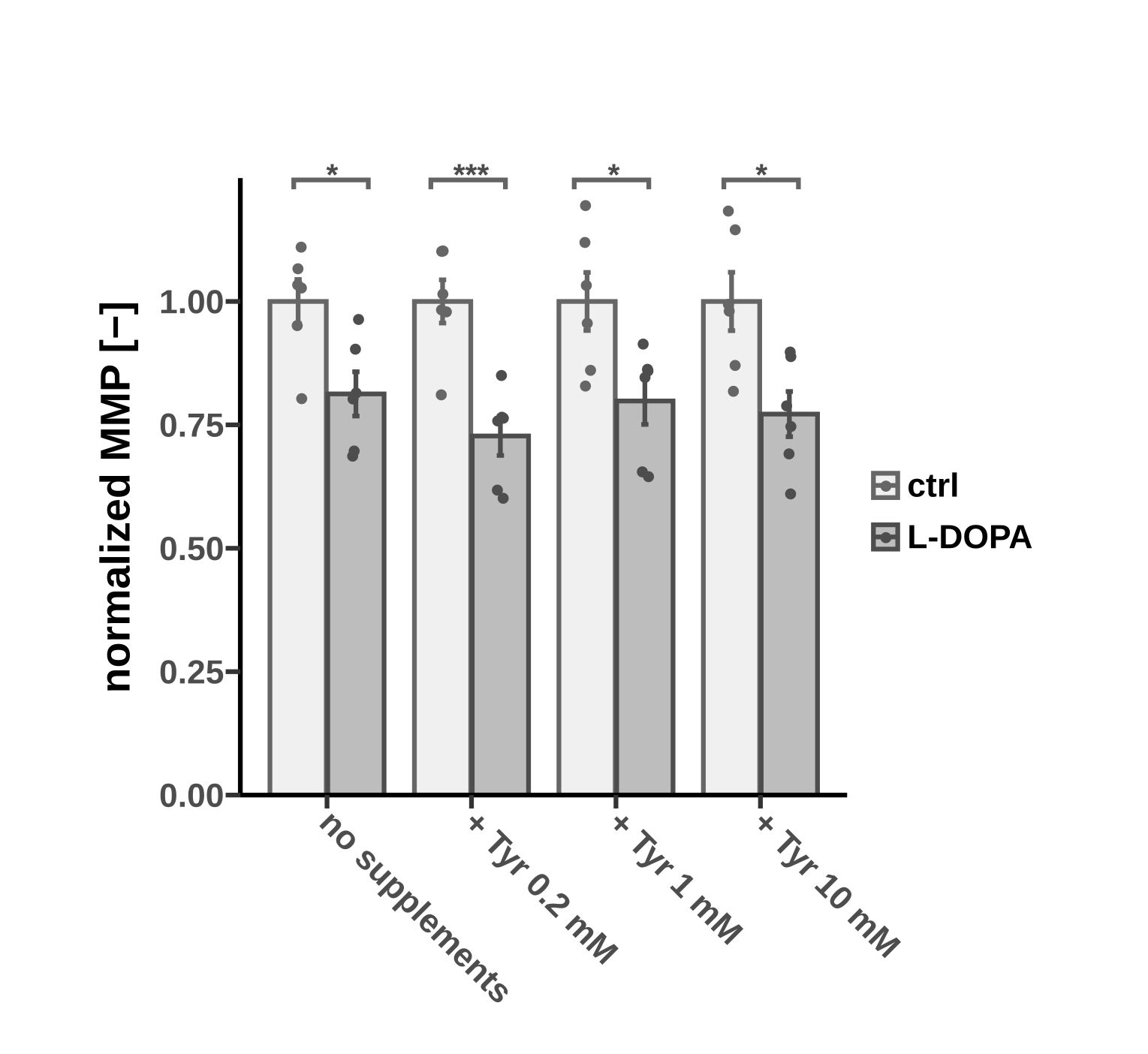

Supplement: Supplementary file 6 — Supplemented Figure 4 [file 41420_2021_547_MOESM6_ESM.png]

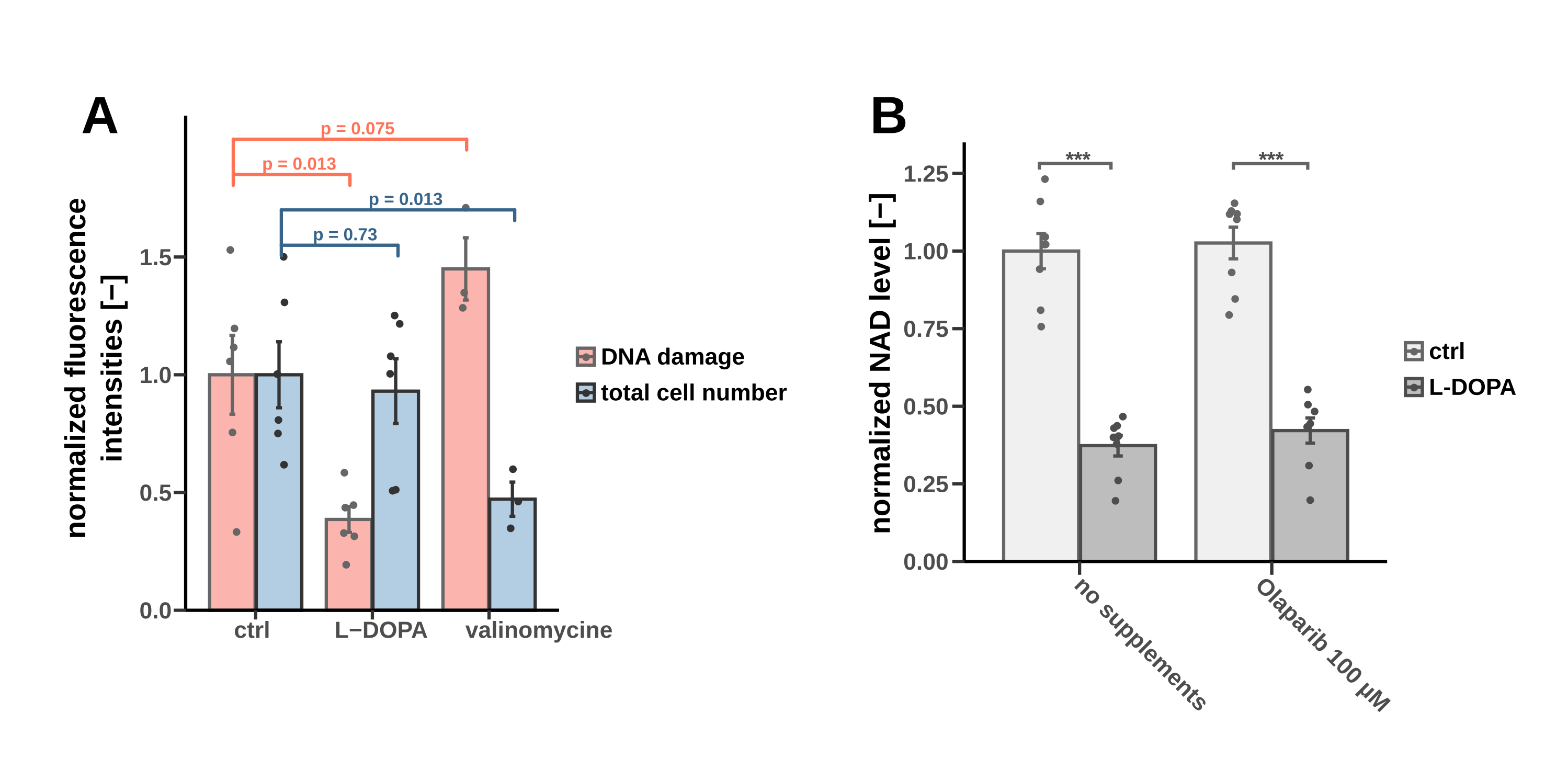

Supplement: Supplementary file 7 — Supplemented Figure 5 [file 41420_2021_547_MOESM7_ESM.png]

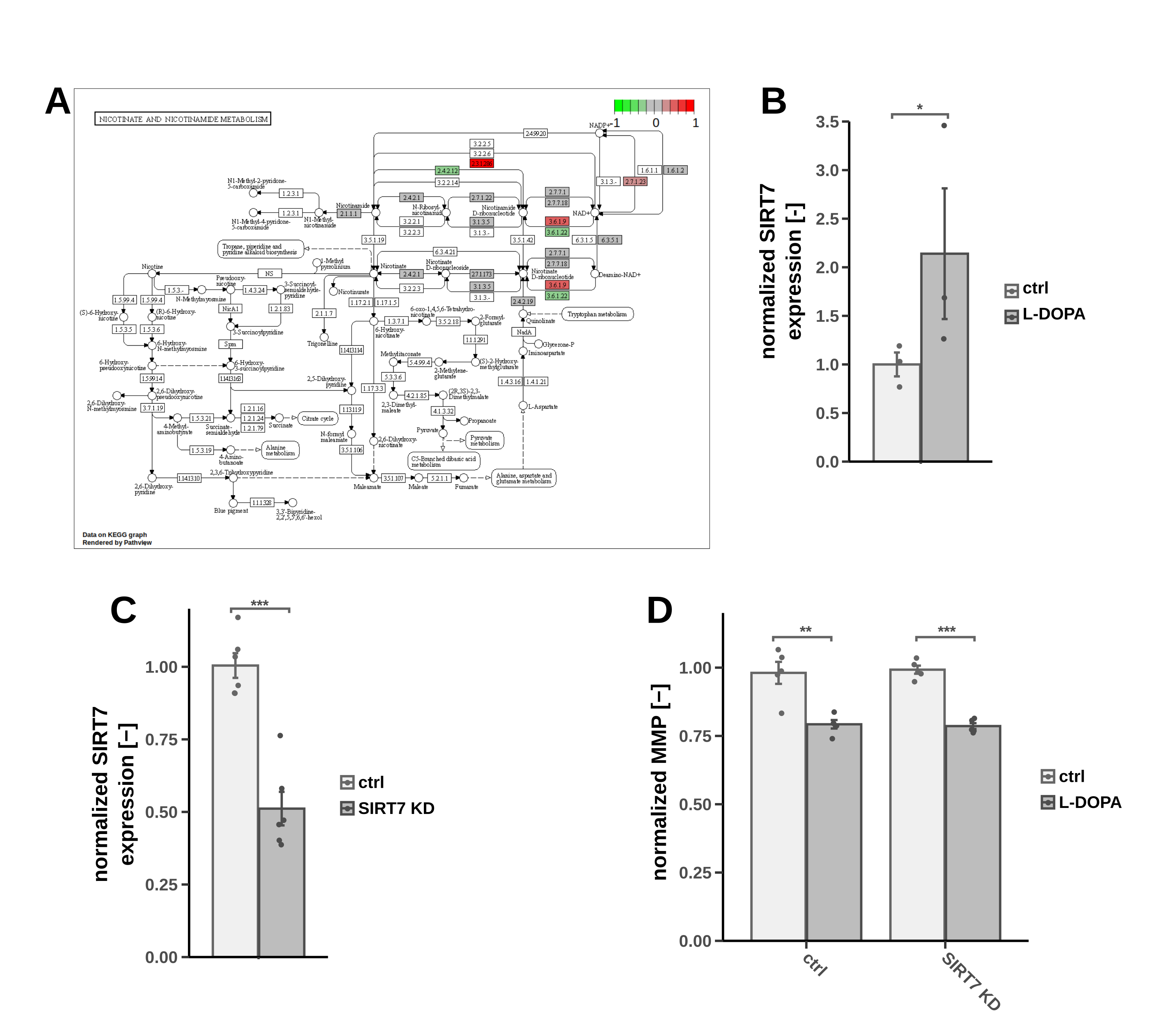

Supplement: Supplementary file 8 — Supplemented Figure 6 [file 41420_2021_547_MOESM8_ESM.png]

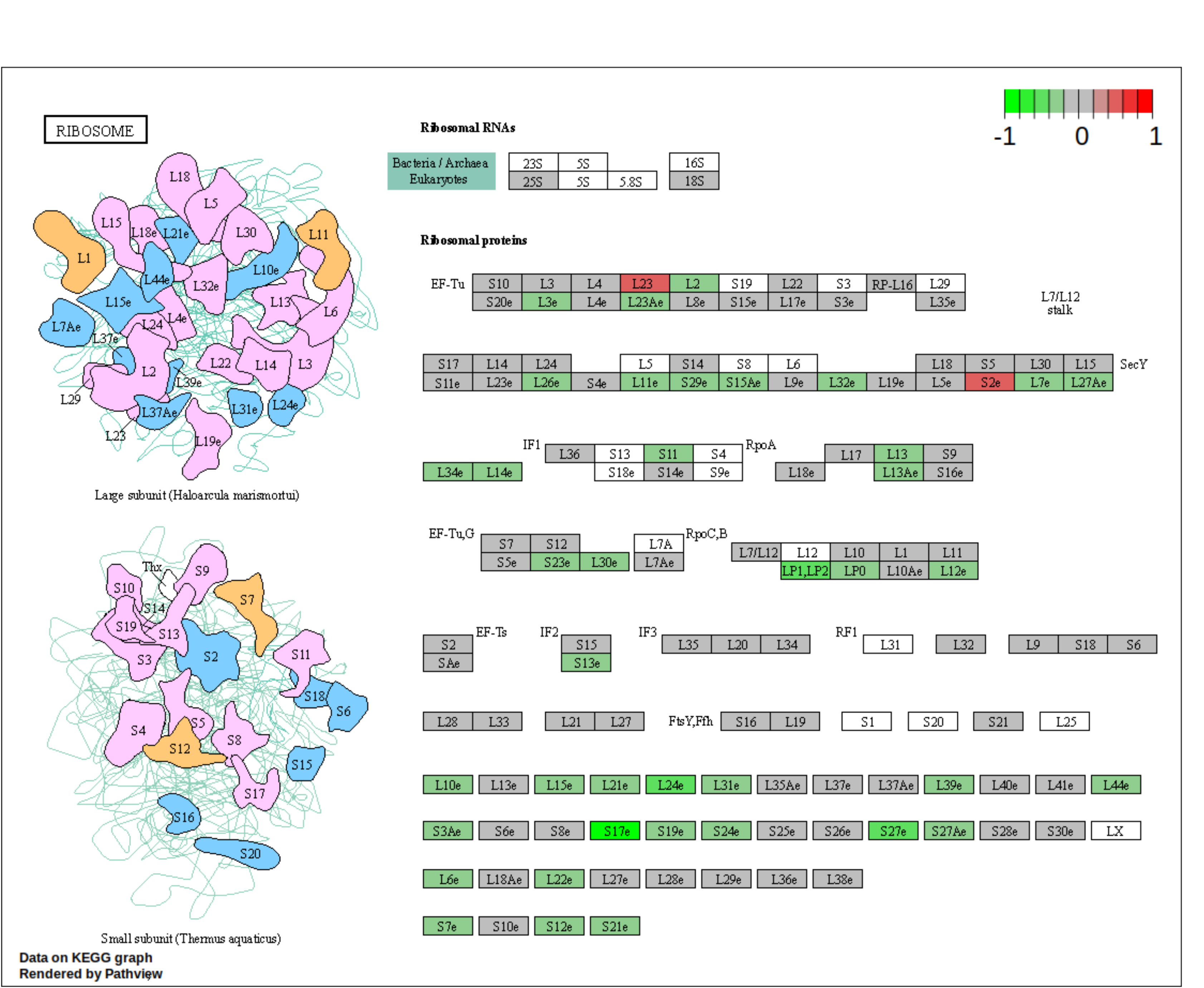

Supplement: Supplementary file 9 — Supplemented Figure 7 [file 41420_2021_547_MOESM9_ESM.png]

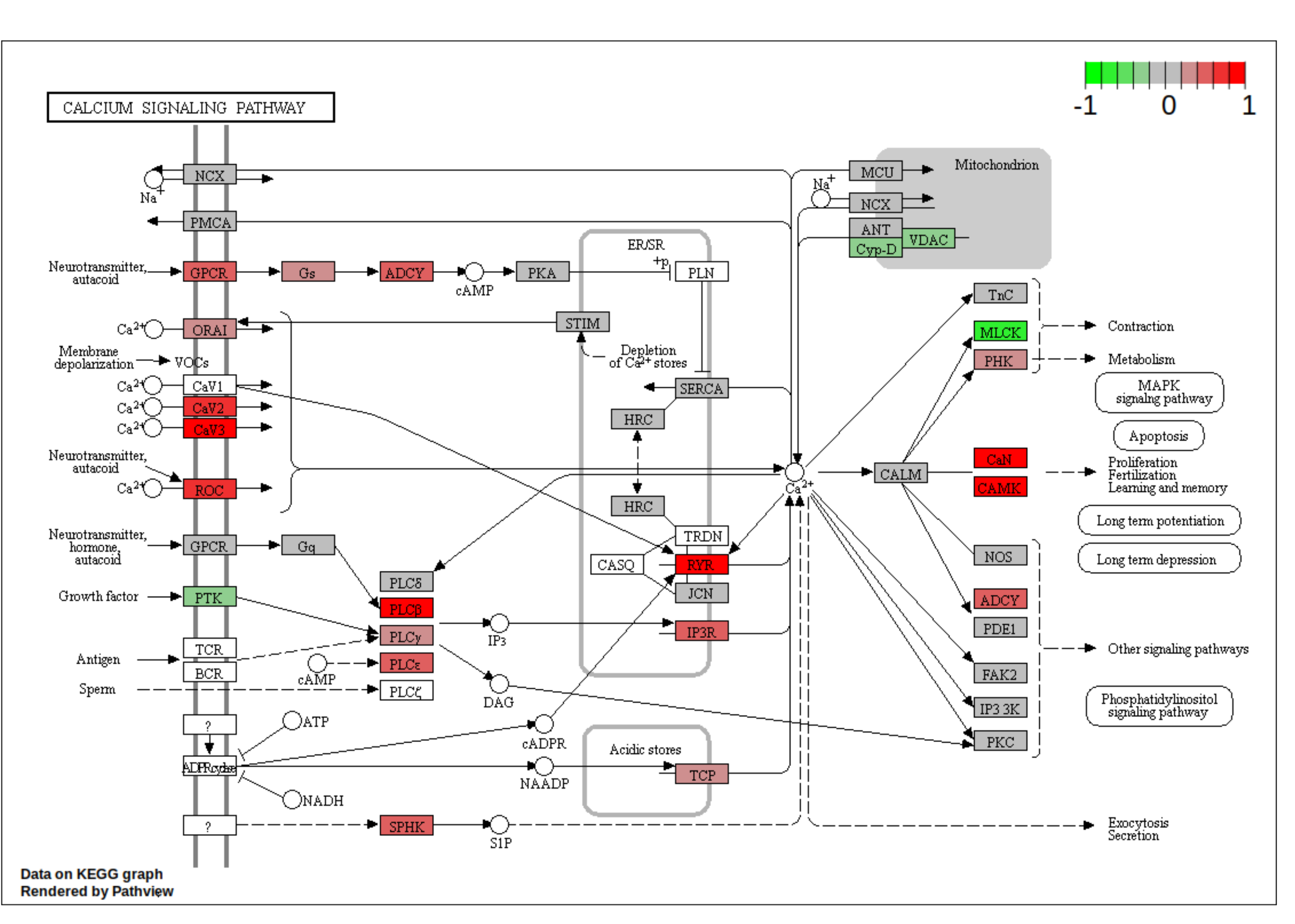

Supplement: Supplementary file 10 — Supplemented Figure 8 [file 41420_2021_547_MOESM10_ESM.png]

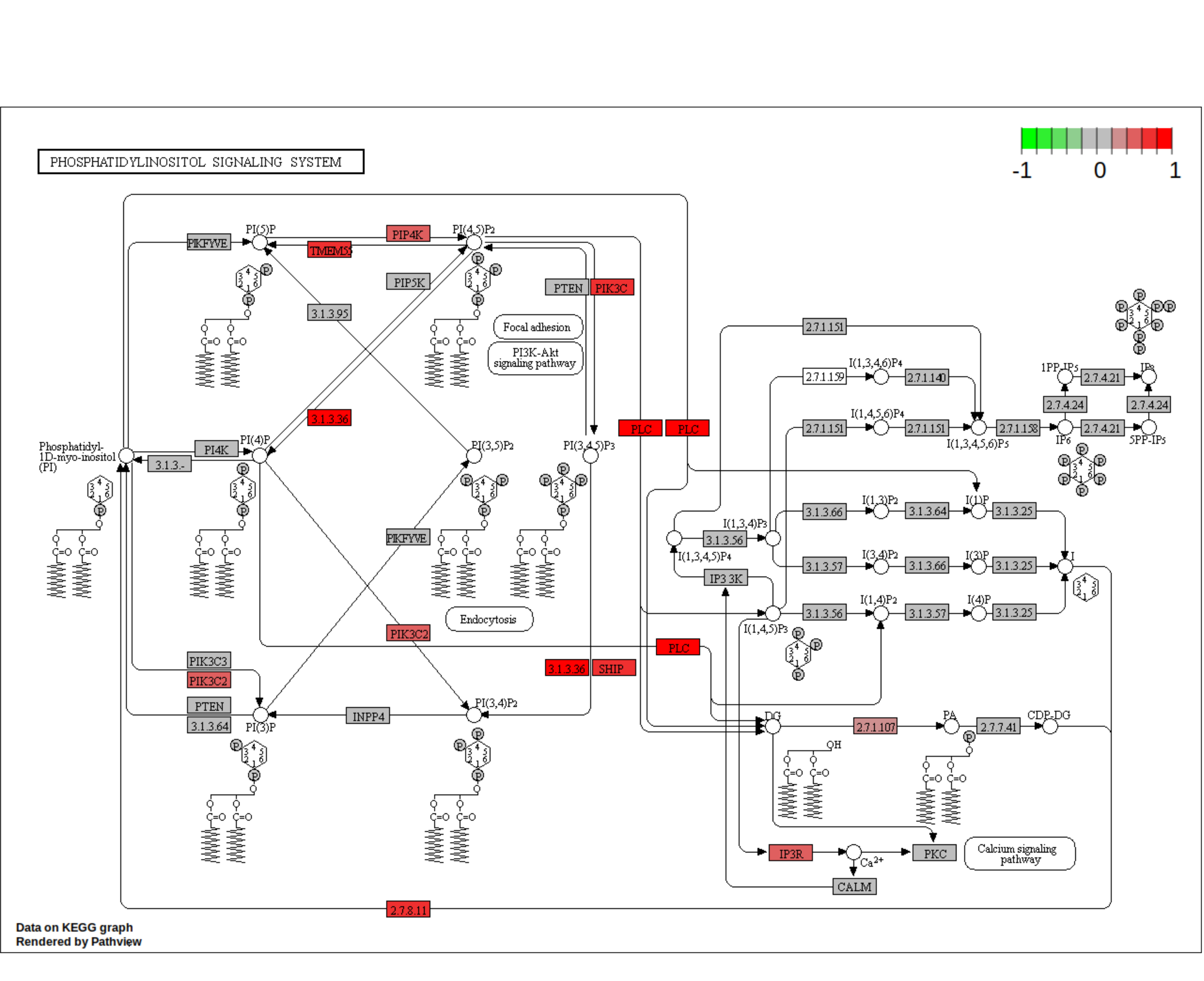

Supplement: Supplementary file 11 — Supplemented Figure 9 [file 41420_2021_547_MOESM11_ESM.png]
